# Supplementary material for: Augmenting Vacuolar H+-ATPase Function Prevents Cardiomyocytes from Lipid-Overload Induced Dysfunction
Source: Int J Mol Sci. 2020 Feb 23;21(4):1520. doi: 10.3390/ijms21041520 (PMC7073192; doi:10.3390/ijms21041520)
Supplement: Supplementary file 1 [file ijms-21-01520-s001.pdf]

Supplemental Figures

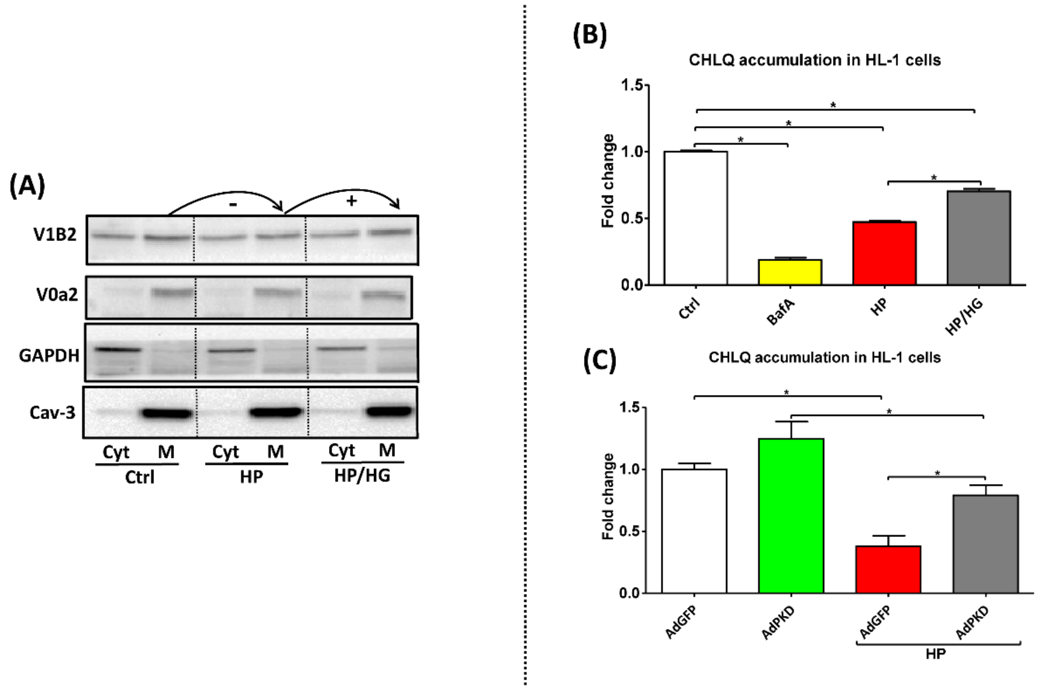

Supplemental Figure. 1 (Related to Figure 1). Status and activity of v-ATPase in lipid-overexposed cardiomyocytes.

(A) Fractionation in HL-1 cells: HL-1 cells were incubated for 24h with either control (Ctrl) medium, HP medium media containing 500µM palmitate and 100nM insulin, or HP medium with 25mM glucose addition (HP/HG). Contents of v-ATPase subunit a2 (V_0-a2) and v-ATPase subunit B2 (V_1-B2) were assessed by western blotting in the cytoplasmic fraction (Cyt) and the membrane fraction (M). Caveolin-3 and GAPDH were detected as the loading control of V_0-a2 and V_1-B2, respectively. Representative blots of three independent experiments are shown.

(B-C) Chloroquine (CQ) accumulation in lipid-overexposed HL-1 cells: **(B)** HL-1 cells were incubated for 24h with either Ctrl medium, Ctrl medium containing 100nM Bafilomycin-A (BafA), HP medium, or HP/HG medium (n=3). **(C)** HL-1 cells were incubated for 32h with either Ctrl medium containing 120µl Adenovirus Green Fluorescent Protein (AdGFP), Ctrl medium containing 120ul Adenovirus protein kinase D1 (AdPKD), HP medium containing 120µl AdGFP (AdGFP/HP), or HP medium containing 120µl AdPKD (AdPKD/HP). After the culturing of all conditions above, cells were ready for [^{3}H] CQ accumulation assay last 20 min. Values are displayed as mean ± SEM (n=3). $^*p<0.05$ were considered statistically significant.

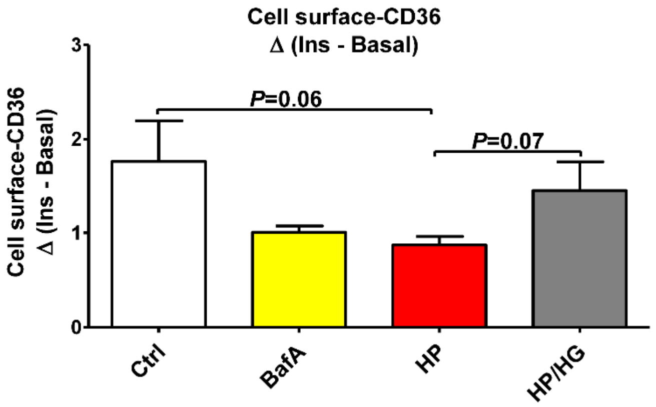

Supplemental Figure. 2 (Related to Figure 2A). The effect of insulin is expressed as difference between acute insulin-stimulated and basal for each condition (Δ Ins-Basal). Values are displayed as mean ± SEM (n=3). $^{*}p<0.05$ were considered statistically significant.

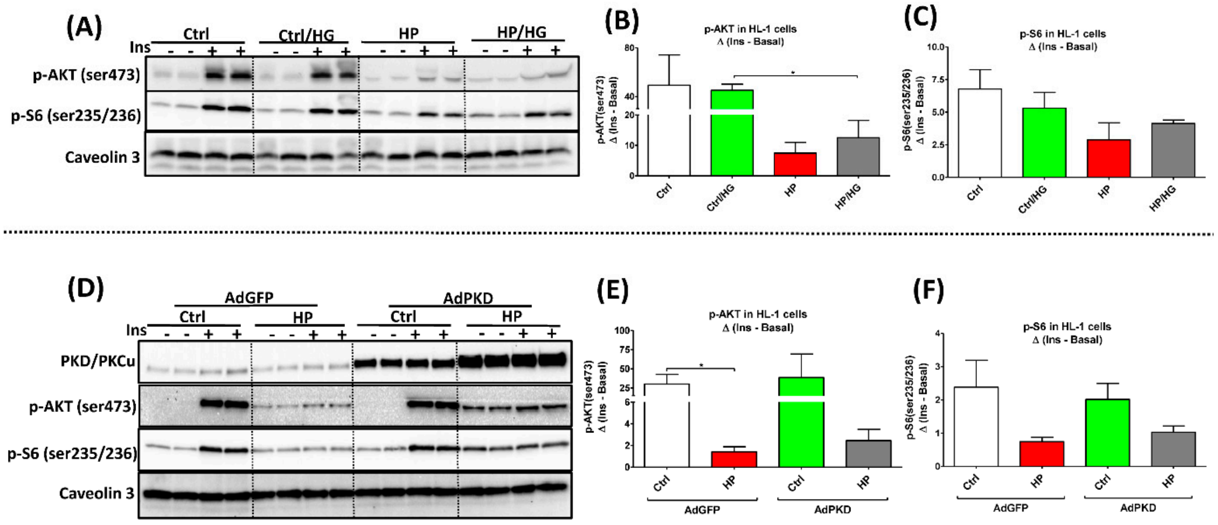

Supplemental Figure. 3 (Related to Figure 3). Insulin sensitivity in lipid overloaded HL-1 cells.

(A-C) HL-1 cells were treated with control (Ctrl) medium, Ctrl medium containing 25mM glucose (Ctrl/HG), HP medium media containing 500μM palmitate and 100nM insulin, and HP medium with 25mM glucose addition (HP/HG) for 24h. Subsequently, cells were stimulated either with or without 200 nM insulin for 30 min and harvested for western blotting analysis of phosphorylation of Akt (pAkt) and of ribosomal protein S6 (pS6). **(A)** Representative blots. **(B-C)** Quantification of the level of pAKT and pS6 (n=3).

(D-F) HL-1 cells were treated with Ctrl medium containing 120μl Adenovirus Green Fluorescent Protein (AdGFP), HP medium containing 120μl AdGFP (AdGFP/HP), Ctrl medium containing 120ul Adenovirus protein kinase D1 (AdPKD), and HP medium containing 120μl AdPKD (AdPKD/HP) for 24h. Subsequently, cells were stimulated either with or without 200 nM insulin for 30 min and harvested for western blotting analysis of pAkt and pS6. **(D)** Representative blots. **(E-F)** Quantification of the level of pAKT and pS6. Values are displayed as mean ± SEM (n=3). $^{*}p<0.05$ were considered statistically significant.

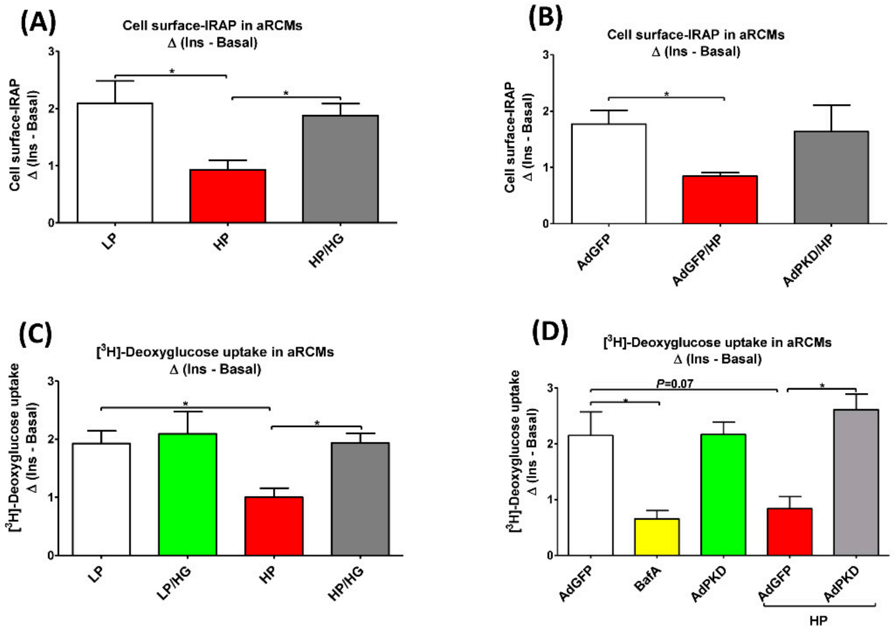

Supplemental Figure. 4 (Related to Figure 4). The effect of insulin is expressed as difference between acute insulin-stimulated and basal for each condition (Δ Ins-Basal).

(A-B) These data are from the same set of experiments as the data from **Figure 4B and D** (n=4).

(C-D) These data are from the same set of experiments as the data from **Figure 4E-F**. Values are displayed as mean ± SEM (n=4). $^{*}p$<0.05 were considered statistically significant.

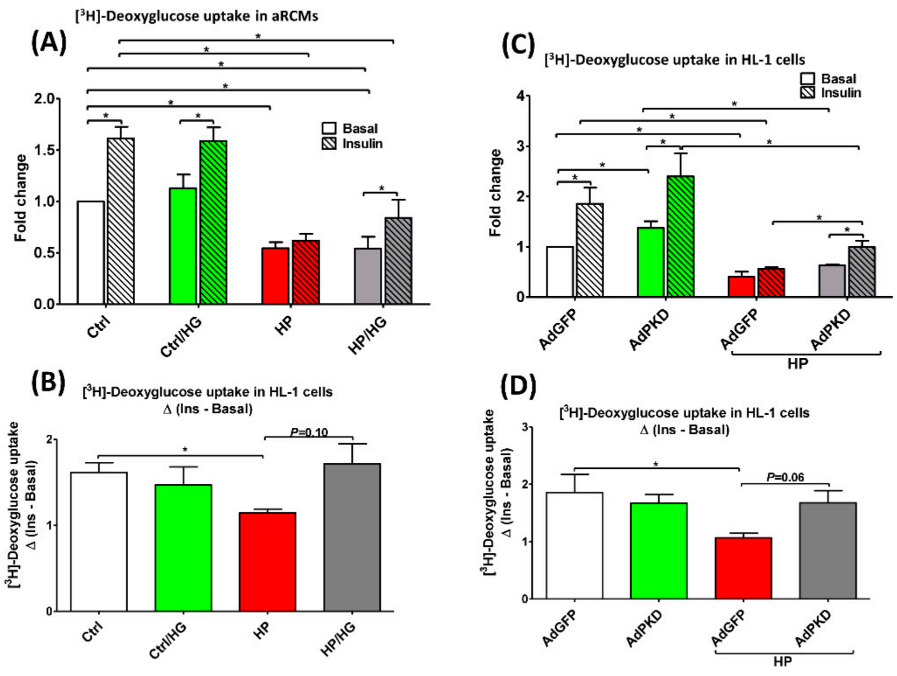

Supplemental Figure. 5 (Related to Figure 4). Insulin-stimulated glucose uptake in lipid-overexposed HL-1 cells.

(A) HL-1 cells were incubated for 24h with control (Ctrl) medium, Ctrl medium supplemented with either 25mM glucose (Ctrl/HG), HP medium media containing 500µM palmitate and 100nM insulin, or HP medium with 25mM glucose addition (HP/HG), followed by 30 min of (-/+) insulin (200 nM) incubation prior to [^{3}H]deoxyglucose labeling. **(B)** The effect of insulin is expressed as difference between acute insulin-stimulated and basal for each condition (Δ Ins-Basal). These data are from the same set of experiments as the data from **Supplemental Fig. 5A** (n=5).

(C) HL-1 cells were incubated for 48h with either Ctrl medium containing 120µl Adenovirus Green Fluorescent Protein (AdGFP), Ctrl medium containing 120ul Adenovirus protein kinase D1 (AdPKD), HP medium containing 120µl AdGFP (AdGFP/HP), or HP medium containing 120µl AdPKD (AdPKD/HP), followed by 30 min of (-/+) insulin (200 nM) incubation prior to [^{3}H] deoxyglucose labeling. **(D)** The effect of insulin is expressed as difference between acute insulin-stimulated and basal for each condition (Δ Ins-Basal). These data are from the same set of experiments as the data from **Supplemental Fig. 5C**. Values are displayed as mean ± SEM (n=4). $^*p<0.05$ were considered statistically significant.

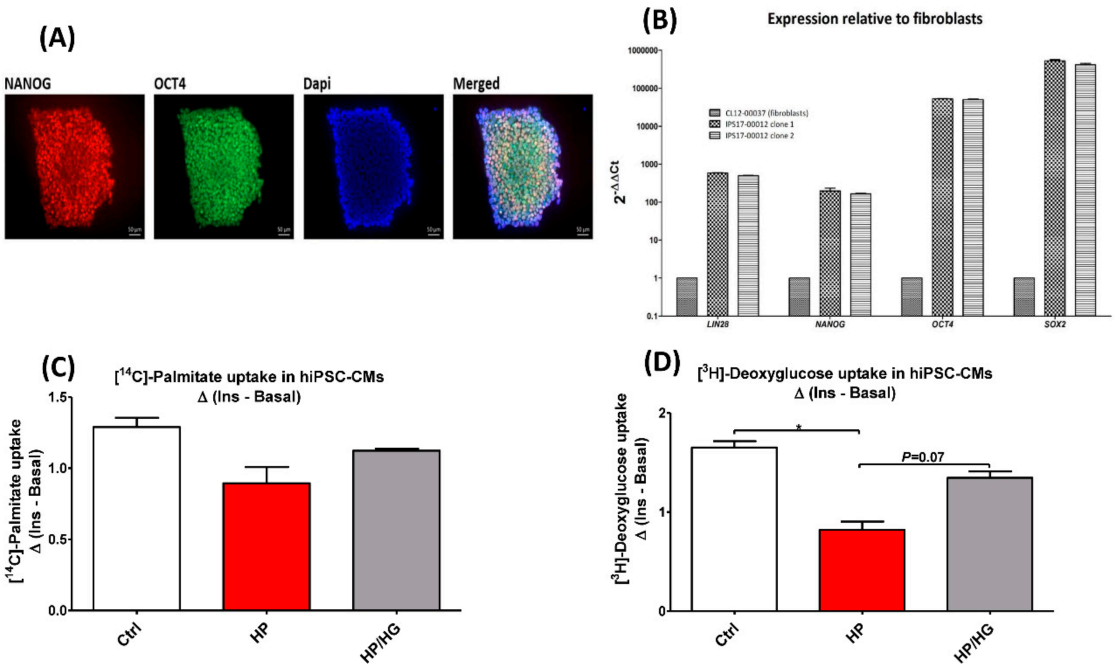

Supplemental Figure. 6: Related to Figure 6.

(A) Expression of pluripotency markers (e.g., Nanog and Oct4) and Dapi in human induced pluripotent stem cells (hiPSCs) by using immunofluorescent staining. Scale bar is 50µm. **(B)** Gene expression of pluripotency markers in hiPSCs relative to fibroblasts.

(C-D) The effect of insulin is expressed as difference between acute insulin-stimulated and basal for each condition (Δ Ins-Basal). These data are from the same set of experiments as the data from **Figure 6B-C**. Values are displayed as mean ± SEM (n=3). $^{*}p<0.05$ were considered statistically significant.

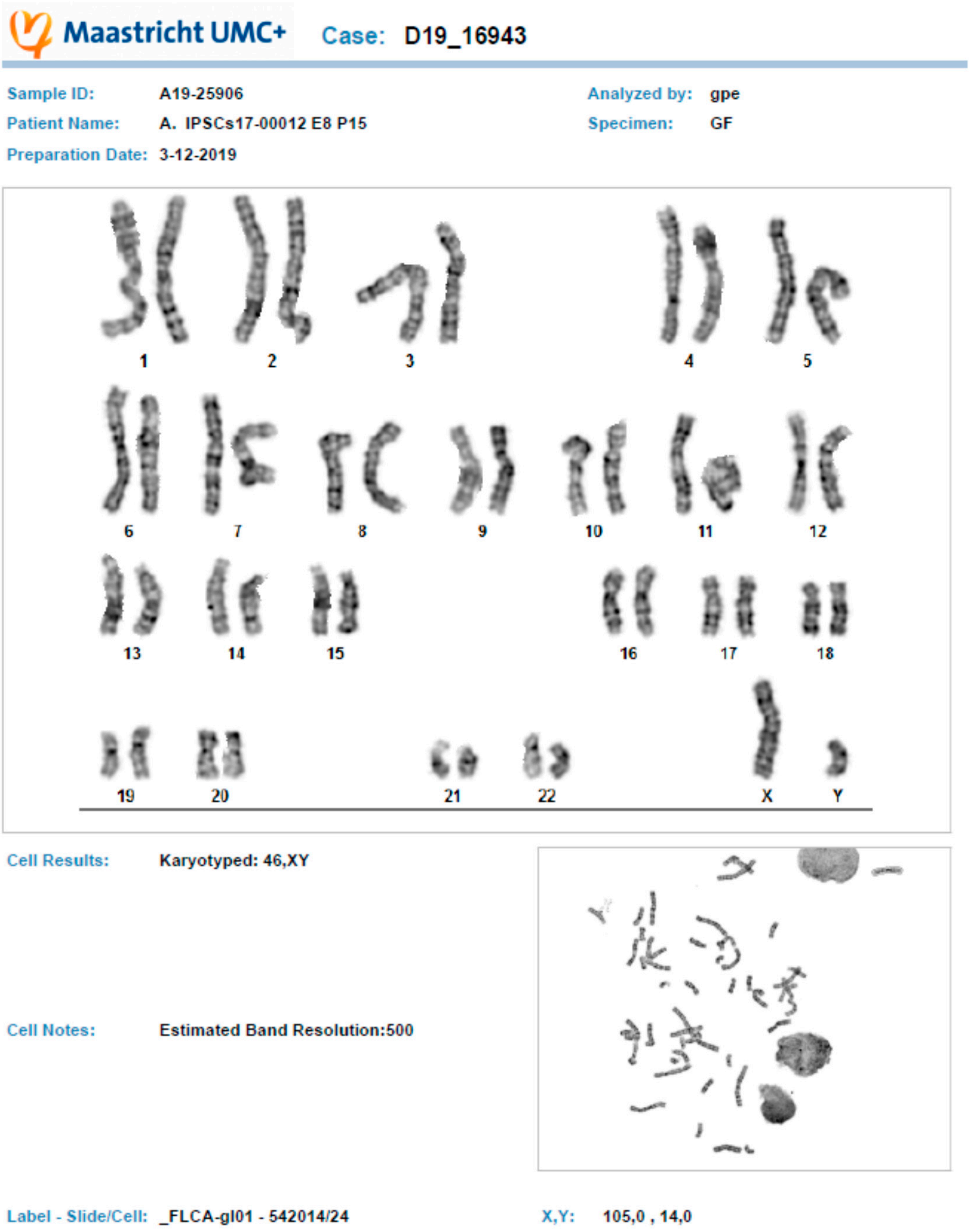

Supplemental Figure. 7: Karyotyping results of hiPSCs from the Department of Clinical Genetics, Maastricht UMC+, demonstrating that there were no chromosomal abnormalities observed in these cells.
